# Celiac disease: a comprehensive current review

**DOI:** 10.1186/s12916-019-1380-z

**Published:** 2019-07-23

**Authors:** Giacomo Caio, Umberto Volta, Anna Sapone, Daniel A. Leffler, Roberto De Giorgio, Carlo Catassi, Alessio Fasano

**Affiliations:** 1 Department of Medical Sciences, University of Ferrara, Via Aldo Moro 8, Cona, 44124 Ferrara, Italy; 2 Center for Celiac Research and Treatment, Massachusetts General Hospital, Boston, MA 02114 USA; 3 Department of Medical and Surgical Sciences, University of Bologna, 40138 Bologna, Italy; 4 Takeda Pharmaceuticals International Co, Cambridge, MA 02139 USA; 5 Division of Gastroenterology, Beth Israel Deaconess Medical Center, Boston, MA 02115 USA; 6 Department of Pediatrics, Center for Celiac Research, Università Politecnica delle Marche, 60121 Ancona, Italy

**Keywords:** Alternative treatment, Clinical phenotypes, Epidemiology, Genetics, Gluten-free diet, Histopathological findings, Pathogenesis, Serological markers

## Abstract

**Background:**

Celiac disease remains a challenging condition because of a steady increase in knowledge tackling its pathophysiology, diagnosis, management, and possible therapeutic options.

**Main body:**

A major milestone in the history of celiac disease was the identification of tissue transglutaminase as the autoantigen, thereby confirming the autoimmune nature of this disorder. A genetic background (*HLA-DQ2/DQ8* positivity and non-HLA genes) is a mandatory determinant of the development of the disease, which occurs with the contribution of environmental factors (e.g., viral infections and dysbiosis of gut microbiota). Its prevalence in the general population is of approximately 1%, with female predominance. The disease can occur at any age, with a variety of symptoms/manifestations. This multifaceted clinical presentation leads to several phenotypes, i.e., gastrointestinal, extraintestinal, subclinical, potential, seronegative, non-responsive, and refractory. Although small intestinal biopsy remains the diagnostic ‘gold standard’, highly sensitive and specific serological tests, such as tissue transglutaminase, endomysial and deamidated gliadin peptide antibodies, have become gradually more important in the diagnostic work-up of celiac disease. Currently, the only treatment for celiac disease is a life-long, strict gluten-free diet leading to improvement in quality of life, ameliorating symptoms, and preventing the occurrence of refractory celiac disease, ulcerative jejunoileitis, and small intestinal adenocarcinoma and lymphoma.

**Conclusions:**

The present review is timely and provides a thorough appraisal of various aspects characterizing celiac disease. Remaining challenges include obtaining a better understanding of still-unclear phenotypes such as slow-responsive, potential (minimal lesions) and seronegative celiac disease. The identification of alternative or complementary treatments to the gluten-free diet brings hope for patients unavoidably burdened by diet restrictions.

## Introduction

Celiac disease (CD) is an autoimmune condition characterized by a specific serological and histological profile triggered by gluten ingestion in genetically predisposed individuals [1]. Gluten is the general term for alcohol-soluble proteins present in various cereals, including wheat, rye, barley, spelt, and kamut [1]. In recent years, there have been significant changes in the diagnosis, pathogenesis, and natural history of this condition [2], with CD undergoing a true ‘metamorphosis’ due to the steady increase in the number of diagnoses identified, even in geriatric patients [2]. This has been mainly attributed to the greater availability of sensitive and specific screening tests, which allow identification of the risk groups for CD and led to a significant raise in diagnoses worldwide [2–5]. Several theories have suggested that the globalization and ubiquitous spread of ‘false’ or ‘extreme’ versions of the Mediterranean diet including the consumption of very high quantities of gluten (up to 20 g/day), has led to an increased prevalence and incidence of CD [3, 4]. In addition, the quality of gluten itself might also play a contributory role. Indeed, the production of new grain variants due to technological rather than nutritional reasons may have influenced the observed increase in the number of CD diagnoses in recent years [4, 5]. However, these hypotheses have not been confirmed and the real cause of the risk in CD diagnoses remains unknown. Furthermore, the epidemiological observation that similar ‘epidemics’ are reported for other autoimmune diseases in the Western hemisphere [6] suggests that environmental factors other than gluten can be at play.

In this article, we aimed to provide a thorough review on the multifaceted features of CD spanning from its epidemiological, pathogenetic, clinical, and diagnostic aspects to therapeutic strategies using a practical approach in order to help general practitioners, internal medicine physicians, and gastroenterologists in their clinical practice.

## Epidemiology

CD is one of the most common autoimmune disorders, with a reported prevalence of 0.5–1% of the general population (Table 1), with the exception of areas showing low frequency of CD-predisposing genes and low gluten consumption (e.g., sub-Saharan Africa and Japan) [7–13]. Studies have shown that most CD cases remain undetected in the absence of serological screening due to heterogeneous symptoms and/or poor disease awareness. CD prevalence is increasing in Western countries. Between the years 1975 and 2000, CD prevalence increased 5-fold in the US, for reasons that are currently unknown [14]. The prevalence of CD is higher in first-degree CD relatives (10–15%) and in other at-risk groups, particularly patients with Down syndrome, type 1 diabetes, or IgA deficiency [1].

**Table 1:** Serological screening for celiac disease in adults (confirmed with duodenal biopsy) in the general population

	First level antibody test	No. of cases	Age, years	Country	Prevalence of celiac disease
Corazza et al., 1997 [6]	EmA	2237	20–87	Italy	0.18%
Ivarsson et al., 1999 [7]	EmA	1894	25–74	Sweden	0.53%
Riestra et al., 2000 [8]	EmA	1170	14–89	Spain	0.26%
Volta et al., 2001 [9]	EmA	3483	14–65	Italy	0.57%
Mustalahti et al., 2010 [10]	Anti-tTG, EmA	6403	30–93	Finland	2.5%
Rubio-Tapia et al., 2012 [11]	Anti-tTG, EmA	7798	23–66	USA	0.71%
Singh et al., 2016 [12]	Anti-tTG, EmA	43,955	Not specified	Asia	0.5%

*Anti-tTG* anti-transglutaminase antibodies, *EmA* anti-endomysium antibodies

## Pathophysiology

CD is a unique autoimmune disease in that its key genetic elements (human leukocyte antigen (HLA)-DQ2 and HLA-DQ8), the auto-antigen involved (tissue transglutaminase (tTG)), and the environmental trigger (gluten) are all well defined. A major drawback in CD research has been the lack of a reliable and reproducible animal model, with the possible exception of the Irish setter dog, which may develop a gluten-related disease [15]. Nevertheless, new technologies pertinent to human gut biology and immunology are opening unprecedented opportunities for major research breakthroughs.

As with many other autoimmune diseases, we have witnessed an epidemic of CD, questioning the previous paradigm that gluten is the only key element dictating the onset of the disease in genetically at-risk subjects. Improved hygiene and lack of exposure to various microorganisms also have been linked with a steep increase in autoimmune disorders in industrialized countries during the past 40 years [1, 16]. The hygiene hypothesis argues that the rising incidence of many autoimmune diseases may partially be the result of lifestyle and environmental changes that have reduced our exposure to pathogens. With breakthroughs in the role of the gut microbiological ecosystem [17] in dictating the balance between tolerance and immune response leading to autoimmunity, this hypothesis is under scrutiny. Regardless of whether autoimmune diseases are due to too much or too little exposure to microorganisms, it is generally accepted that adaptive immunity and imbalance between T helper 1 and 2 cell responses are key elements of the pathogenesis of the autoimmune process. Besides genetic predisposition and exposure to gluten, loss of intestinal barrier function, a pro-inflammatory innate immune response triggered by gluten, inappropriate adaptive immune response, and an imbalanced gut microbiome all seem to be key ‘ingredients’ of the CD autoimmunity recipe.

### Genetics

As with any other autoimmune disease, CD has a strong hereditary component as testified by its high familial recurrence (~ 10–15%) and the high concordance of the disease among monozygotic twins (75–80%) [18]. Also common to other autoimmune diseases is the relevant role of HLA class II heterodimers, specifically DQ2 and DQ8, in the heritability of CD. HLA-DQ2 homozygosis confers a much higher risk (25–30%) of developing early-onset CD in infants with a first-degree family member affected by the disease [19–21]. Since HLA-DQ2/HLA-DQ8 is frequent among the general population (25–35%), and only 3% of these HLA-compatible individuals will go on to develop CD [22], it is not surprising that genome-wide association studies have identified more than 100 non-HLA-related genes associated with CD [18, 23]. The relevance of these additional genes in conferring genetic risk for CD is rather limited, but they may lead to the discovery of key pathways potentially involved in disease pathogenesis.

### Gluten as an environmental trigger of CD

Introduced 10,000 years ago during the transition from a nomadic lifestyle to agricultural settlements, gluten-containing grains are a recent addition to the human diet. Moreover, gluten is one of the few digestion-resistant proteins consumed chronically in significant quantities and is constituted by several non-digestible immunogenic peptides. These two characteristics could help in breaking the tolerance to this food antigen, when the immune system is activated, as can happen during an enteric infection. Gliadins, key components of gluten, are complex proteins unusually rich in prolines and glutamines and are not completely digestible by intestinal enzymes [24]. The final product of this partial digestion is a mix of peptides that can trigger host responses (increased gut permeability and innate and adaptive immune response) that closely resemble those instigated by the exposure to potentially harmful microorganisms [25–28].

### Gluten trafficking from lumen to lamina propria (paracellular and transcellular)

**Fig. 1:**
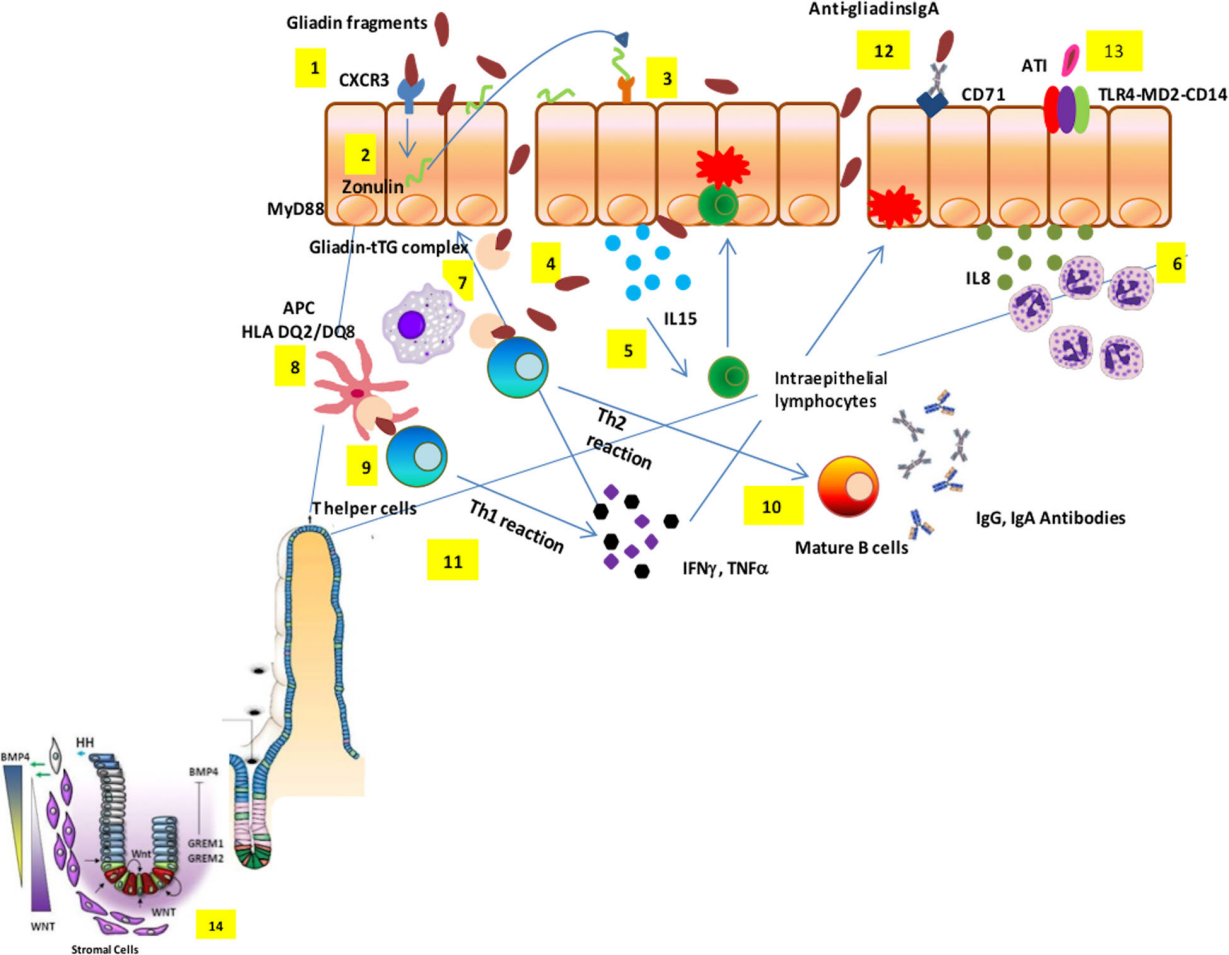
Celiac disease pathogenesis. Partially digested gliadin fragments interact with chemokine receptor 3 on the apical side of epithelium (1) inducing a myeloid differentiation primary response 88-dependent release of zonulin (2). Zonulin interacts with the intestinal epithelium and triggers increased intestinal permeability (3). Functional loss of the gut barrier facilitates gliadin peptide translocation from lumen to the lamina propria (4). Gliadin peptides trigger release of IL-15, keratinocyte growth factor, and IL-8 (5), with consequent recruitment of neutrophils in the lamina propria (6). Simultaneously, alpha-amylase/trypsin inhibitors engage the Toll like receptor 4–MD2–CD14 complex with subsequent up-regulation of maturation markers and release of proinflammatory cytokines (7). Following innate immune-mediated apoptosis of intestinal cells with subsequent release of intracellular tissue transglutaminase, gliadin peptides are partially deamidated (8). Deamidated gliadin is recognized by DQ2/8^+^ antigen presenting cells (9) and then presented to T helper cells (10). T helper cells trigger activation and maturation of B cells, producing IgM, IgG, and IgA antibodies against tissue transglutaminase (11). T helper cells also produce pro-inflammatory cytokines (interferon γ and tumor necrosis factor α) (12), which in turn further increase gut permeability and, together with T killer cells, initiate the enteropathy. Damaged enterocytes express CD71 transporter also on their apical side, resulting in retrotranscytosis of secretory IgA-gliadin complexes (13), thus potentiating gluten trafficking from gut lumen to lamina propria. Ultimately, the interaction between CD4^+^ T cells in the lamina propria with gliadin induces their activation and proliferation, with production of proinflammatory cytokines, metalloproteases, and keratinocyte growth factor by stromal cells, which induces crypt hyperplasia and villous blunting secondary to intestinal epithelial cell death induced by intraepithelial lymphocytes. The hyperplastic crypts (14) are characterized by an expansion of the immature progenitor cells compartment (WNT) and downregulation of the Hedgehog signaling cascade. An increased number of stromal cells known to be part of the intestinal stem cell niche and increased levels of bone morphogenetic protein antagonists, like Gremlin-1 and Gremlin-2, may further contribute to the crypt hyperplasia present in celiac disease

Studies from our group and others have shown that gliadin can cause an immediate and transient increase in intercellular tight junction permeability of intestinal epithelial cells [23, 24] (Fig. 1). This effect has been linked to the release of zonulin, a family of molecules that increases paracellular permeability by causing tight junction disassembly [29–31]. Gliadin enhances zonulin-dependent increased gut paracellular permeability irrespective of disease status [32–39]. Similarly, when tested in C57BL/6 mice duodenal tissues, gliadin caused a myeloid differentiation primary response 88-dependent increase in gut mucosa permeability [40]. We have also identified two alpha-gliadin motifs that can modulate the intestinal barrier function by binding to chemokine receptor 3, with subsequent zonulin release that causes disassembly of the interepithelial tight junction complex [41]. The involvement of the paracellular pathway for gluten trafficking in the lamina propria has also been corroborated by genetic studies identifying an association of some tight junction genes with CD [42–44]. There is solid evidence that gluten can also cross the intestinal barrier through the transcellular pathway once tolerance to gluten has been broken [45, 46]. The transferrin receptor CD71, normally expressed on the basolateral side of enterocytes, is overexpressed on the luminal side of the intestinal epithelium in CD patients during the acute phase of the disease, leading to an apical-to-basal retrotranscytosis of gliadin peptides complexed with secretory IgA [47]. This retrotranscytosis of secretory IgA–gliadin complexes protects gliadin fragments from lysosomal degradation and promotes the entry of harmful gliadin peptides into the intestinal lamina propria [47], thereby perpetuating intestinal inflammation initiated by the paracellular passage of these peptides (Fig. 1). Because of their resistance, the gluten immunogenic peptides (GIP) can cross the defective epithelial lining, reach the blood stream (thus extending the inflammatory process), and finally be excreted with the urine [48].

### The innate immune response

Innate immunity plays a critical role in initiating CD, and cytokines such as interleukin (IL)-15 and interferon α can prime the innate immune response by polarizing dendritic cells and intraepithelial lymphocyte function [49]. Recent results suggest that specific gliadin peptides may induce epithelial growth factor and an IL-15-dependent proliferation of enterocytes, structural modifications, vesicular trafficking alterations, signaling and proliferation, and stress/innate immunity activation [50]. Alpha-amylase/trypsin inhibitors – molecules conferring pest resistance in wheat – also seem to play a key role in CD innate immune response by engaging the Toll-like receptor 4–MD2–CD14 complex with subsequent up-regulation of maturation markers and release of proinflammatory cytokines in cells from CD patients [51]. These mucosal events, along with the functional breach of epithelial barrier function secondary to the gliadin-mediated zonulin release [29–36], the subsequent access of toxic peptides in the lamina propria, and gliadin-induced production of high levels of the neutrophil-activating and chemoattractant chemokine IL-8 [26, 52], cause the ‘perfect storm’ to initiate CD enteropathy (Fig. 1). More recently, our group showed that gliadin exerts a direct neutrophil chemoattractant effect by interacting with fMet-Leu-Phe receptor 1 [53, 54].

### The adaptive immune response

The erroneous adaptive immune response consequence of a highly specific interplay between selected gluten peptides and major histocompatibility complex class II HLA-DQ2/8-antigen restricted T cells plays a paramount role in CD pathogenesis [55]. Dependent on the post-translational deamidation of gluten peptides by transglutaminase 2 (TG2), this interplay is influenced by the initial imprinting of the innate immune system through IL-15 upregulation that promotes the CD4^+^ T cell adaptive immune response [56, 57]. Presentation of gluten to CD4^+^ T cells carried out by dendritic cells as well as macrophages, B cells, and even enterocytes expressing HLA class II, can cause their recirculation in the lamina propria [58]. The contact of CD4^+^ T cells in the lamina propria with gluten induces their activation and proliferation, with production of proinflammatory cytokines, metalloproteases, and keratinocyte growth factor by stromal cells, which induces cryptal hyperplasia and villous blunting secondary to intestinal epithelial cell death induced by intraepithelial lymphocytes (IELs) [58]. Additionally, there is an overexpression of membrane-bound IL-15 on enterocytes in active CD causing over-expression of the natural killer (NK) receptors CD94 and NKG2D by CD3^+^ IELs [59]. CD crypt hyperplasia has been hypothesized to be the consequence of an imbalance between continuous tissue damage due to the mucosal autoimmune insult described above and inability of the stem cells to compensate. We have recently provided a more mechanistic, evidence-based explanation for hyperplastic crypts in active CD by showing that the celiac hyperplastic crypt is characterized by an expansion of the immature progenitor cell compartment and downregulation of the Hedgehog signaling cascade [60]. These data shed light on the molecular mechanisms underlying CD histopathology and illuminate the reason for the lack of enteropathy in the mouse models for CD. Indeed, lack of consistent CD-like enteropathy in humanized mice [61] supports the concept that the accelerated disruption of enterocytes secondary to the adaptive CD4^+^ T cell insult cannot fully explain CD pathogenesis, supporting the notion that an intrinsic defect of the stem cell compartment in subjects at risk of CD is a key element of CD enteropathy [60, 62].

### The role of the gut microbiome in the pathogenesis of CD

In Western countries, a rise in the overall prevalence of CD has been well documented, but the reasons for this ‘epidemic’ remain elusive. The combination of epidemiological, clinical, and animal studies suggests that broad exposure to a wealth of commensal, non-pathogenic microorganisms early in life are associated with protection against CD and that pre-, peri-, and post-natal environmental factors may strongly influence the gut ecosystem [17]. Therefore, the hygiene hypothesis concept can be misleading, while an ‘environment-dependent dysbiosis hypothesis’ would more closely reflect the interplay between host and environmental pressure dictating the balance between health and disease. Several studies have shown an association between CD and a change in the microbiome composition [63, 64]. However, these associative studies do not necessarily imply causation between microbiota composition and CD pathogenesis. Many environmental factors known to influence the composition of the intestinal microbiota are also thought to play a role in the development of CD [19, 21].

It has been reported that, compared to control infants, neonates at family risk of CD had a decreased representation of Bacteriodetes and a higher abundance of Firmicutes [65]. This study also showed that infants who developed autoimmunity had decreased lactate signals in their stools coincident with a diminished representation in *Lactobacillus* species in their microbiome, which preceded the first detection of positive antibodies [65]. Early microbiota alterations in infants were also suggested in a recent study comparing microbial communities between DQ2^+^ and DQ2^−^ infants [66]. However, to move from association to causation, large-scale, longitudinal studies are necessary to define if and how gut microbiota composition and metabolomic profiles may influence the loss of gluten tolerance and subsequent onset of CD in genetically susceptible subjects.

### Clinical presentation

CD is diagnosed more frequently in women with a female-to-male ratio ranging from 2:1 to 3:1 [1, 2]. However, based on serological screening, the actual female-to-male ratio is 1.5:1 [67]. The disease can occur at any age from early childhood to the elderly, with two peaks of onset – one shortly after weaning with gluten in the first 2 years of life, and the other in the second or third decades of life. The diagnosis of CD can be challenging since symptoms can vary significantly from patient to patient [68].

In 2011, the Oslo classification of CD identified the following clinical presentations: classic, non-classic, subclinical, potential and refractory [69]. Instead of the ‘classic/non-classic’ categorization, which does not fully reflect current clinical presentations, in this review, we will use a more practical terminology, i.e., intestinal/extraintestinal. These two terms better represent the main clinical phenotypes of CD, which may occur individually (i.e., intestinal vs. extraintestinal) or in combination [70].

The intestinal form of CD is more commonly detected in the pediatric population and children younger than 3 years and is characterized by diarrhea, loss of appetite, abdominal distention, and failure to thrive [71]. Older children and adults may complain of diarrhea, bloating, constipation, abdominal pain, or weight loss [72]. Nonetheless, in adults, the malabsorption syndrome with chronic diarrhea, weight loss and significant asthenia is quite rare. Despite its uncommon detection, this phenotype can cause hospitalization due to cachexia, sarcopenia, significant hypoalbuminemia, and electrolyte abnormalities. Conversely, an irritable bowel syndrome (IBS)-like presentation with constipation or alternating bowel and/or dyspepsia-like symptoms, such as nausea and sometimes vomiting, is more frequent [2].

Extraintestinal symptoms are common in both children and adults [2, 72]. They include iron deficiency microcytic anemia, detectable in up to 40% of cases (by cause of iron malabsorption or chronic inflammation) [73] or, more rarely, macrocytic anemia due to folic acid and/or vitamin B12 deficiency (more frequent in Europe than in the US). Changes in bone mineral density, including osteopenia or osteoporosis (affecting about 70% of patients at diagnosis), are related to altered absorption of calcium and vitamin D3 [74]. In children, growth retardation and short stature can raise the suspect of an underlying CD. Other signs include tooth enamel defects, aphthous stomatitis (identified in about 20% of undiagnosed CD patients) [75], and hypertransaminasemia (40–50% of untreated patients), which can be ascribed to food and bacterial antigen translocation reaching the liver due to increased intestinal permeability [76]. A wide array of neurological symptoms, such as headache, paresthesia, neuroinflammation, anxiety and depression, can be detectable in CD patients. The clinical presentation may also include changes in reproductive function characterized by late menarche, amenorrhea, recurrent miscarriages, premature birth, early menopause, and changes in the number and mobility of spermatozoa. Notably, these manifestations can be reversed when patients start a strict gluten-free diet (GFD), although fatigue and some neurological manifestation as well as functional gastrointestinal (GI) symptoms can persist for a long period in a subgroup of CD patients [2, 77–81].

**Fig. 2:**
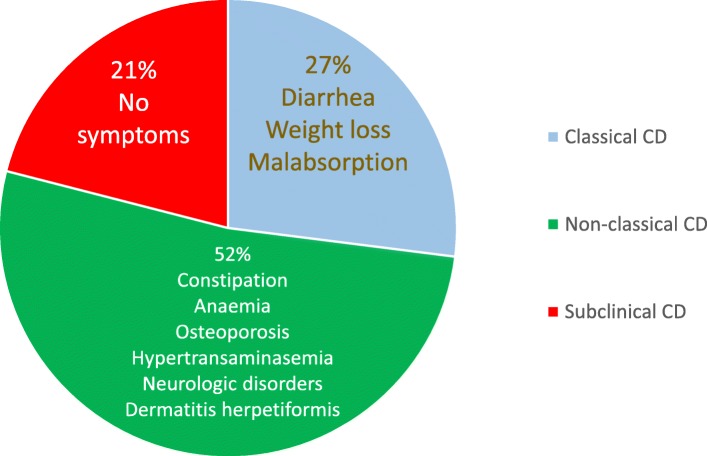
Prevalence of clinical phenotypes of adult celiac disease in our experience

The subclinical form includes patients with symptoms/signs below the clinical identification threshold and are often recognizable only after the appreciation of the beneficial effects induced by the GFD. A typical example of subclinical cases are those patients undergoing antibody screening due to being relatives of CD patients or cases identified as a result of a screening strategy in the general population [2, 69]. The prevalence of various CD clinical phenotypes observed in our experience is reported in Fig. 2.

CD can be associated with different autoimmune and idiopathic diseases, including dermatitis herpetiformis (which, as a single manifestation, should prompt testing for CD), type 1 diabetes mellitus, Hashimoto’s thyroiditis, selective IgA deficiency, alopecia areata, Addison’s disease, connective tissue diseases (mainly Sjogren’s syndrome), chromosomal diseases (Down, Turner, and William’s syndromes), neurological diseases (cerebellar ataxia, peripheral neuropathy, epilepsy with and without occipital calcifications), hepatic autoimmune diseases (primary biliary cholangitis, autoimmune hepatitis, primary sclerosing cholangitis), and idiopathic dilated cardiomyopathy (Table 2) [2, 82–93]. The importance of diagnosing CD associated with these concomitant diseases is twofold since a GFD is able to resolve symptoms, prevent complications, and improve some of the CD associated diseases [2].

**Table 2:** Diseases associated with celiac disease

Autoimmune	Idiopathic	Chromosomal
Type 1 diabetes mellitus	Dilated cardiomyopathy	Down syndrome
Hashimoto’s thyroiditis	Epilepsy with or without occipital calcifications	Turner syndrome
Graves’ disease	Cerebellar ataxia	William’s syndrome
Autoimmune hepatitis	Peripheral neuropathy	
Primary biliary cholangitis	Multiple myoclonic seizures	
Primary sclerosing cholangitis	Multiple sclerosis	
Dermatitis herpetiformis	Cerebral atrophy	
Vitiligo	Chronic inflammatory intestinal diseases	
Addison’s disease	Sarcoidosis	
Alopecia	Atopy	
Psoriasis		
IgA deficiency		
Autoimmune atrophic gastritis		
Autoimmune hemolytic anemia		
Sjogren’s syndrome		
Scleroderma		
Systemic erythematosus lupus		
Polymyositis		
Rheumatoid arthritis		
Myasthenia gravis		
IgA nephropathy (Berger’s disease)		

The potential form of CD is characterized by positive serological and genetic markers with a normal intestinal mucosa and minimal signs of inflammation such an increase in IELs [69]. Patients with the potential form can manifest with classic and non-classic symptoms or be entirely asymptomatic. The scientific community has not universally agreed on whether or not a GFD should be prescribed for patients with potential CD.

Finally, refractory CD (RCD) is characterized by persistent symptoms and atrophy of the intestinal villi after at least 12 months of a strict GFD. RCD can lead to complications such as ulcerative jejunoileitis, collagenous sprue, and intestinal lymphoma [69].

**Fig. 3:**
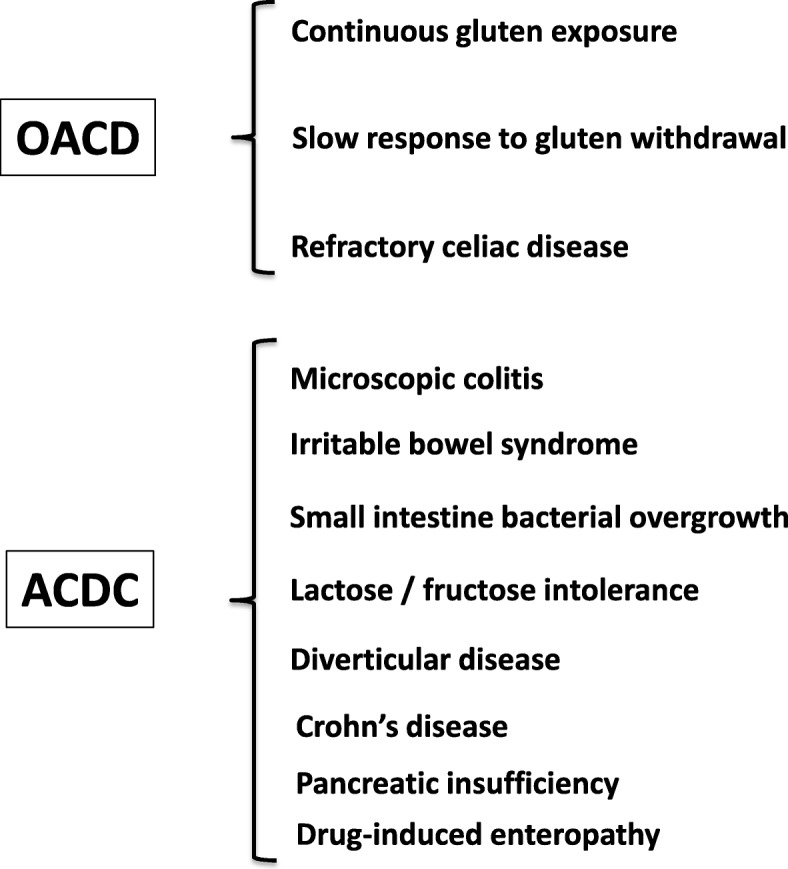
Causes of ongoing signs and/or symptoms of celiac disease (CD) despite a gluten-free diet (formerly referred to as ‘non-responsive’ CD). In this review, two clinical phenotypes have been proposed – ongoing active celiac disease (OACD), related to three main causes, and associated celiac disease conditions (ACDC), encompassing a wide array of diseases

In recent years, other forms of CD (not included in the Oslo Classification [69]), i.e., seronegative and GFD non-responsive CD, have been identified in the clinical practice. The seronegative form is characterized by the lack of demonstrable serological markers along with clinical signs of severe malabsorption and atrophy of the intestinal mucosa [94]. This form should be included in the differential diagnosis with other diseases that cause atrophy of the intestinal villi. The term non-responsive CD indicates GI symptoms that persist despite a GFD of more than 12 months [95]; however, it does not differentiate between active CD and associated conditions, which can be responsible for symptom persistence (Fig. 3) and alternative terminology is discussed below.

## Diagnosis

The gold standard for CD diagnosis is represented by the combination of mucosal changes detected by duodenal biopsy and by positivity of serological tests (anti-tTG antibodies, anti-endomysium antibodies (EmA), and deamidated gliadin peptide (DGP) antibodies). Despite the progress made in serology, no antibody test currently available provides a sensitivity and specificity of 100% (Table 3) [96, 97], thus requiring intestinal biopsy as a key adjunct for establishing a correct diagnosis [98]. Pediatric patients with high titers (over 10 times the cut-off) of anti-tTG antibodies, detectable EmA, HLA-DQ2/HLA-DQ8 positivity, and signs/symptoms suggestive of CD may skip duodenal biopsy as recommended by recent guidelines by the European Society for Paediatric Gastroenterology Hepatology and Nutrition (ESPGHAN) [99]. Although a large multicenter European study showed diagnostic accuracy of ESPGHAN criteria in identifying CD in children [100], it should be pointed out that these criteria are not followed worldwide. In fact, in some countries such as the USA, ESPGHAN criteria are not recommended because of the poor reproducibility of the anti-tTG assays [101]. Both advantages and disadvantages exist to biopsy for children with suspected celiac disease; however, most pediatric cases, especially those with low to medium anti-tTG2 titers, require histopathological assessment to confirm celiac disease diagnosis. In a recent study, Fuchs et al. [102] showed that the combination of anti-tTG (over 10 times the cut-off), EmA, and HLA-DQ2/HLA-DQ8 positivity (triple criteria) had a good accuracy across the range of pre-test probabilities in detecting adult patients with CD. Nonetheless, duodenal biopsy still represents a pillar in the diagnosis of adult patients with suspected CD.

**Table 3:** Performance of serological markers for a diagnosis of celiac disease

	Sensitivity (%)	Specificity (%)	PPV (%)	NPV (%)	Diagnostic accuracy (%)
Anti-tTG IgA	96.8	91.0	91.2	96.8	97.7
EmA IgA	93.7	100	100	94.4	96.9
DGP IgG	84.4	98.5	98.2	86.8	91.6

*Anti-tTG* anti-transglutaminase antibodies, *DGP* direct antibodies against deamidated gliadin peptides, *EmA* anti-endomysium antibodies, *NPV* negative predictive value, *PPV* positive predictive value

Current standard of care is based on the “*four out of five rule*” [103], which indicates that four out of five of the following criteria are enough to establish CD diagnosis: (1) typical signs and symptoms (diarrhea and malabsorption); (2) antibody positivity; (3) HLA-DQ2 and/or HLA-DQ8 positivity; (4) intestinal damage (i.e., villous atrophy and minor lesions); and (5) clinical response to GFD. Additionally, this rule helps physicians to identify the various subtypes of CD, i.e., seronegative CD (absence of point 2), potential CD (absence of point 4), non-classic CD (absence of point 1), and non-responsive CD (absence of point 5).

### Hematology and blood biochemistry tests

Routine blood tests can lead to suspect CD [104]. Low serum levels of hemoglobin, albumin, calcium, potassium, magnesium, and phosphorus are more commonly detected in CD with a classic rather than non-classic phenotype. Most patients develop an iron deficiency microcytic anemia with low ferritin values. Normocytic, macrocytic, or dimorphic anemia is less common in CD patients with an increased variability in the size of red blood cells due to concomitant malabsorption of folate and/or vitamin B12, particularly in cases associated with autoimmune atrophic gastritis [73]. Elevated levels of bone-specific alkaline phosphatase and a significant vitamin D3 deficiency can be found in patients with CD and osteopenia/osteoporosis [105]. A cryptogenic increase of transaminases may herald the presentation of CD even in the absence of other relevant symptoms. Notably, transaminases revert to normal within 6–12 months of a GFD [76]. In a moderate percentage of adult CD patients, a blood smear can detect changes in the membrane and cytoplasm of red blood cells (i.e., Howell–Jolly bodies), whereas pitted red cells can be identified by Nomarski phase contrast microscopy; both these red blood cell abnormalities suggest an underlying hyposplenism [106]. Another sign of hyposplenism is the detection of a marked thrombocytosis in association with a small (in the most severe cases even undetectable) spleen revealed by ultrasound. Macroscopically evident or even functional (no major changes at imaging) hyposplenism is a predisposing factor for the development of infectious diseases due to encapsulated bacteria (e.g., Pneumococcus, Meningococcus), and is associated with autoimmune diseases and complications such as refractory CD, ulcerative jejunoileitis, and lymphoma [107, 108].

### Serology

Over the last 20 years, the routine use of serological tests led to a significant increase in CD diagnoses. CD-related antibodies can identify subjects with suspected CD, further confirmed by histological evaluation [98]. In the early 1980s, anti-gliadin antibodies were the first serological marker used to screen patients at risk for CD. However, due to their low specificity, this serological test has been dismissed and its role is now confined to the possible identification of a subset of cases with non-celiac gluten/wheat sensitivity [109]. Currently, the serological diagnosis of CD is based on tests that are highly predictive and widely validated, including EmA, anti-tTG, and DGP [97]. CD-related antibodies belong to IgA and IgG classes, but only those of IgA class can be regarded as highly sensitive and specific for CD [97]. The use of IgG markers (except for DGP) is often misleading due to the high percentage of false positives, and their use should be limited to patients with IgA deficiency [110]. EmA is the antibody test with the highest diagnostic accuracy since it offers an absolute specificity if tested in third-level laboratories by expert operators [111, 112]. The sensitivity of anti-tTG IgA is higher than that of EmA IgA (97% vs. 94%), while the specificity of tTG IgA is certainly lower than that of EmA (91 and 99%, respectively) (Table 3) [96]. False positives for anti-tTG normally display a low antibody titer (less than twice the cut off). A transient positivity for anti-tTG IgA, not associated with duodenal mucosal damage, has been reported in patients with type 1 diabetes at onset followed by a subsequent disappearance of antibodies within 6 months of their identification [113].

**Fig. 4:**
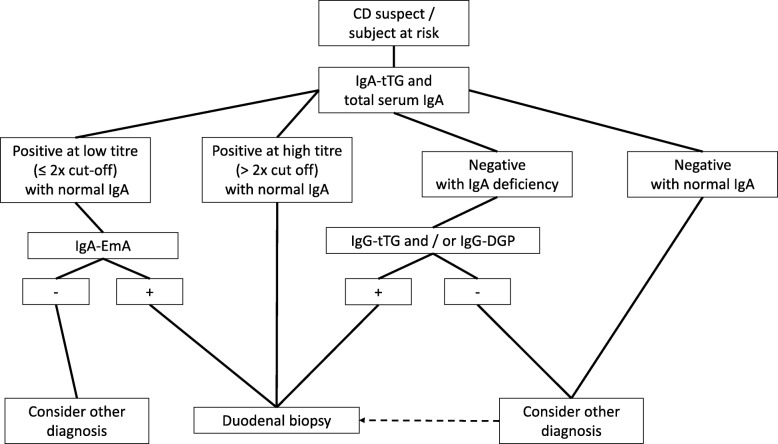
Diagnostic algorithm for celiac disease diagnosis

Another serological marker for CD is represented by DGP [96]. Compared to native peptides, the deamidation of gliadin by tTG makes the modified gliadin peptides more immunogenic. Initial studies reported an elevated sensitivity and specificity for CD [96], although other data showed a decrease in diagnostic accuracy [114]. IgG DGP are particularly useful in identifying CD in early childhood (age < 2 years) [115]. IgA DGP have been shown to be of little usefulness in diagnosing CD and therefore are not recommended for diagnosis [97]. In adult CD, serology should include testing anti-tTG IgA along with total IgA. Should anti-tTG IgA be positive at a high titer with normal total IgA level, a duodenal biopsy can be performed without assessing EmA. With a low titer anti-tTG IgA, EmA IgA testing is necessary and, if positive, a duodenal biopsy should be recommended to confirm CD diagnosis (Fig. 4).

Strict compliance with a GFD in most CD patients leads to the disappearance or significant decrease of antibodies within 12 months (18–24 months if the antibody titer is very high) together with regrowth of the intestinal villi. IgA anti-tTG antibodies are the most commonly used test to monitor CD patients during follow-up, although their disappearance does not reflect the regrowth of intestinal villi [97, 116]. Recent data from Choung et al. [117] demonstrated a very high specificity and sensibility of a new assay directed to identify the serum immune response to epitopes of the tTG-DGP complex. In addition to diagnosis, such markers can be useful for follow-up purposes, although further studies are eagerly needed. While waiting for the validation of a tTG–DGP complex assay, current serology is not enough for evaluating the response to GFD and the regrowth of villi [118, 119].

### Duodenal biopsy

Morphological evaluation of the duodenal biopsy is still of critical importance for confirming CD diagnosis. Histology remains the ‘gold standard’ for CD diagnosis [94]. In recent years, however, the histological criteria for CD have radically changed with the inclusion of mild villous atrophy and minimal lesions (characterized by an isolated increase in IELs) as possible expression of gluten-related intestinal damage [120, 121]. Current recommendations are for four biopsies on the second duodenal portion and two biopsies at the bulb [122]. A fundamental principle for the correct evaluation is the orientation of biopsy samples using cellulose acetate Millipore filters [123, 124]. The different types of CD-related lesions of the intestinal mucosa can be categorized into five stages according to the Marsh classification, modified by Oberhüber, which is currently used in all reference centers for the diagnosis of CD [120]. Type 1 and type 2 lesions, characterized by an increase in IELs (with or without crypt hyperplasia) and normal villi, compatible with, but non-specific for CD. Together with positive anti-tTG and EmA, minimal intestinal lesions indicate potential CD. In most cases, minimal lesions are attributable to other causes, including food allergies (e.g., cow milk proteins), Crohn’s disease, lymphocytic colitis, bacterial and parasitic intestinal infections, such as *Giardia*, common variable immunodeficiency, small intestinal bacterial overgrowth, non-steroidal anti-inflammatory drugs, and *Helicobacter pylori* infection (Box 1) [125–127].

**Fig. 5:**
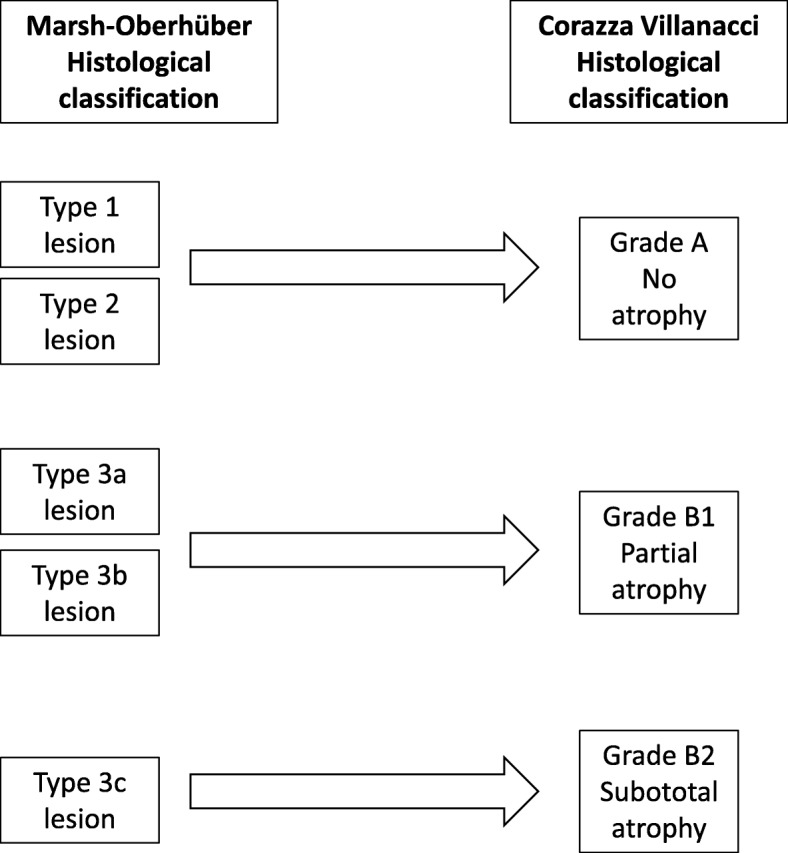
Comparison between the two classifications for the duodenal biopsy

In recent years, there has been a worrying increase in the number of diagnoses of CD incorrectly based on minimal lesions with no genetic and serological markers [128]. The IEL cytometric pattern is more accurate than subepithelial deposits of anti-TG2 IgA for identifying CD in lymphocytic enteritis [129]. The normal IEL cut-off has been established to be ≥25 lymphocytes over 100 epithelial cells. Even if it is well established that coeliac patients always display IEL counts ≥25%, a recent paper stressed the importance of a high IEL count for CD diagnosis underlining that the mean IEL count in untreated CD was 54 ± 18/100 enterocytes, whereas in non-CD patients the value was 13 ± 8 [130]. The typical lesion of CD shows villous atrophy with a change in the villi-to-crypt ratio (< 3:1 to 1:1) and an increase in IEL. This lesion, defined as type 3 in the Marsh–Oberhüber classification, is in turn subdivided into three stages depending on the severity of the atrophy, namely mild (3a), partial (3b), and subtotal atrophy (3c) [120]. Recently, Marsh et al. [131, 132] argued against Oberhüber’s lesion III sub-division, claiming that splitting intestinal atrophy in three stages can be clinically irrelevant and sometimes misleading. In line with this theory no significant difference in IEL count was observed in mild, partial, and subtotal villous atrophy [130]. In an attempt to simplify the histopathological grading and therefore the relationship between pathologists and clinicians, Corazza and Villanacci proposed a classification from five to three stages [121]. Notably, the lesions that characterize CD were divided into two categories – non-atrophic (grade A) and atrophic (grade B) – with the latter being further subcategorized into B1, in which the villi-to-crypt ratio is less than 3:1 (with identifiable villi), and B2, in which villi are entirely atrophic. Grade A lesions, characterized by a pathological increase in the number of IELs, better identified by immunohistochemical staining for CD3, include type 1 and 2 lesions based on the Marsh–Oberhüber classification; grade B1 lesions include the 3a and 3b lesions, while grade B2 corresponds to 3c (Fig. 5) [121]. In some patients with more distal disease or in those with contraindication to biopsy, videocapsule endoscopy can be recommended [133].

## Classification of variants of CD

### Potential CD

In recent years, an increasing number of patients have antibody positivity (IgA EmA and anti-tTG) for CD with HLA-DQ2/HLA-DQ8 and lack of villous atrophy [134, 135]. For this category of patients, which represents around 10% of subjects with CD, the term potential celiac disease has been adopted [69]. In patients with potential CD the intestinal mucosa may be normal (Marsh 0) or slightly inflamed (increased number of IELs, i.e., Marsh 1) [135]. Despite the absence of severe lesions in the intestinal mucosa, these patients may have GI and/or extraintestinal symptoms or be entirely asymptomatic [2, 135]. Although the criteria for diagnosing this condition are clear, potential CD still remains a poorly studied area, with many unsettled questions and contrasting results in the studies conducted so far [135–141]. In children, over 80% of patients with potential CD are asymptomatic and the remaining 20% more commonly experience intestinal symptoms such as malabsorption, chronic diarrhea, and recurrent abdominal pain rather than extraintestinal signs such as iron-deficiency anemia, hypertransaminasemia, and short stature [137, 138, 141]. In adults, however, several studies have shown that the symptomatic phenotype in subjects with potential CD is much more common than in children, and it is primarily characterized by extraintestinal symptoms [135, 136, 139, 140]. One controversial issue concerns whether subjects with potential CD should be treated by a GFD. The actual evidence suggests that a GFD should be recommended only to subjects with symptomatic potential CD. On the other hand, patients with asymptomatic potential CD are allowed to continue a gluten-containing diet while being followed-up with close clinical, serological, and histological control visits (in our experience every 6 months) [135–140]. Studies have reported possible fluctuation with spontaneous normalization of serological markers in patients with potential CD left on a gluten-containing diet. Few patients with potential CD consuming a gluten-containing diet develop full-blown villous atrophy [135, 137, 138, 140, 142]. In our study, only 6% of these subjects developed villous atrophy over a mean follow-up period of 3 years, whereas symptomatic subjects should be treated as they show a clear clinical improvement in symptoms with a GFD [135].

### Seronegative CD

**Fig. 6:**
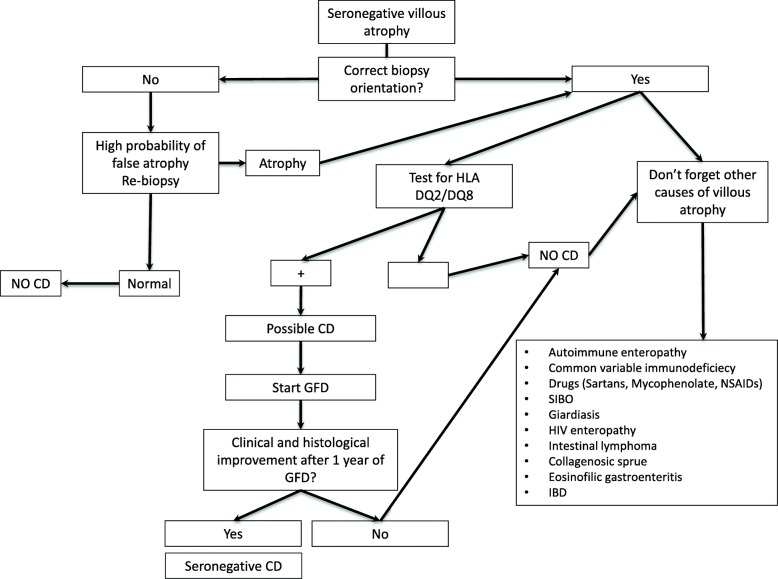
Diagnostic algorithm for seronegative villous atrophy. *SIBO* small intestinal bacterial overgrowth

Although the specific antibodies for CD can be detected in the vast majority of patients, a small number of CD patients (around 2–3%) test negative for serological markers. In these cases, the diagnosis is closely connected to the detection of villous atrophy on the duodenal histology [94, 139, 143]. Performing a genetic test for CD remains a fundamental step since its negative result definitively rules out the disease and prompts physicians to seek for other causes of villous atrophy. A seronegative CD can be confirmed 1 year after the beginning of a GFD, a convenient time to demonstrate an improvement in both symptoms and histology. The diagnostic complexity of this particular variant of CD is due to the differential diagnosis with other conditions involving villous atrophy, such as parasitic infections (*Giardia lamblia*), autoimmune enteropathy, bacterial contamination of the small intestine, common variable immunodeficiency, eosinophilic gastroenteritis, drug-induced enteropathy (angiotensin II receptor antagonists, i.e., olmesartan and other sartans, non-steroidal anti-inflammatory drugs, and mycophenolate), intestinal lymphoma, Crohn’s disease, tropical sprue, HIV enteropathy, and Whipple disease (Fig. 6) [94, 144, 145]. Of all villous atrophies lacking CD antibodies, 28–45% are due to an underlying seronegative CD [94, 146, 147]. Seronegative CD patients display a classic clinical phenotype, characterized by diarrhea and malabsorption, a clear female gender prevalence, and have a higher risk of morbidity and mortality compared with antibody-positive CD patients [94, 147]. Furthermore, compared to classic CD, seronegative patients have a greater association with autoimmune diseases and a higher risk of developing refractory disease. This increased morbidity could be partly due to the late diagnosis of this condition, which on average is around 50 years of age [94].

## Assessment of ongoing signs and symptoms in CD

The majority of the patients with CD exhibit a symptomatic and mucosal response to the GFD. Some patients, however, fail to have complete control of symptoms and normalization of villous structure despite attempted adherence to the GFD. These patients have traditionally been referred to as non-responsive CD [95, 148]; however, this terminology has resulted in confusion as, in many cases, manifestations are due to associated conditions rather than CD. In light of both emerging tests for CD monitoring, such as GIPs, and emerging novel therapies for active CD, we propose updating this classification (formerly non-responsive CD). When evaluating a patient with CD on a GFD and with ongoing signs or symptoms, the initial step is the differentiation between ongoing active CD (OACD) and the presence of associated CD conditions (ACDCs). OACD can be seen in three scenarios – (1) slow response, where there is progressive improvement in symptoms and mucosal damage, but full remission does not occur for at least 1–2 years; (2) RCD, where there is ongoing severe enteropathy and malabsorptive symptoms after 6–12 months on a GFD; and (3) gluten exposure, where, despite adequate understanding of the GFD and attempted adherence, gluten avoidance is insufficient to result in symptomatic or histologic remission. This is the most frequent cause of OACD and can be due to very high sensitivity to a low level of gluten exposure or an inability of the patient to achieve standard recommended gluten restriction. Conversely, when patients with ongoing symptoms are found not to have OACD, generally when small bowel assessment shows minimal ongoing enteropathy and significant gluten exposure is excluded, investigation of possible ACDCs is recommended. ACDCs include IBS, small intestinal bacterial overgrowth, microscopic colitis, lactose intolerance, fructose intolerance, diverticular disease, Crohn’s disease, pancreatic insufficiency, and autoimmune and drug-induced enteropathy, and should be evaluated according to clinical suspicion (Fig. 3) [95, 148].

## CD complications

It has been widely shown that a late diagnosis of CD (after the age of 50) and/or not following a strict GFD can lead to a higher mortality compared to that of the general population [149]. Although rare (around 1% of patients diagnosed with CD) [150], the complications of CD include hyposplenism, RCD, intestinal lymphoma, small bowel adenocarcinoma, and ulcerative jejunoileitis. Complications should be suspected in all patients who, despite adherence to a GFD, complain of an unexplained persistence or re-exacerbation of symptoms (i.e., diarrhea, intestinal sub-occlusion, abdominal pain, weight loss, fever, and severe asthenia). These complications occur more commonly when a diagnosis of CD was established in elderly patients and/or in those who are homozygous for DQ2 not observing a strict GFD [151].

### Hyposplenism

Anatomical or functional hyposplenism can be identified in around 30% of adult patients with CD, with prevalence increasing up to 80% in patients with complications [107, 152]. In CD cases, the detection of a small-size spleen on abdominal ultrasound should guide physicians to confirm functional hyposplenism by evaluating Howell–Jolly bodies (on a peripheral blood smear) or pitted red cells with phase-contrast microscopy (see above) [107, 152]. Splenic hypofunction is closely associated not only with the development of complications and other autoimmune diseases associated with CD but also encapsulated bacterial infections (i.e., *Pneumococcus*, *Haemophilus influenzae*, *Meningococcus*) [107]. Because of the greater risk of developing infections (in some cases lethal or with severe sequelae) from encapsulated bacteria, anti-pneumococcal and anti-meningococcal vaccinations are recommended in this subgroup of patients [106, 107, 152].

### Refractory CD

RCD represents about 10% of all OACD cases [148] and approximately 1–1.5% of total cases of CD [153]. This condition is characterized by symptoms of malabsorption, weight loss, and diarrhea associated with persistent villous atrophy after at least 1 year on a strict GFD, confirmed by negative CD serology [69]. Before thinking of RCD, physicians should rule out other more frequent causes of ongoing signs and symptoms of CD, as previously reported [95, 148]. Refractory CD is in turn subdivided into two categories, primary and secondary, depending on whether the patients had a symptomatic response since the beginning of GFD, or they had a recurrence of symptoms after a more or less long period of improvement.

There are two subtypes of RCD – type 1, where the IEL population has a normal CD3^+^CD8^+^ phenotype, and type 2, with a clonal presentation of surface CD3^−^/intracytoplasmic CD3^+^ IELs along with monoclonal rearrangement of the gamma-chain of the T cell receptor [153]. This distinction into two subtypes is fundamental for therapeutic management and prognosis; in fact, type 2 displays a 5-year mortality rate of 55% vs. 7% for type 1 [154]. The mortality of patients with type 2 RCD is primarily due to the development of intestinal lymphoma, which appears to occur more often in male patients, although CD is more commonly detectable in female patients (female-to-male ratio 3:1) [155]. A diagnosis of RCD should always be suspected by persistent villous atrophy despite a strict, 1-year GFD, negative serology (some cases may show the persistence of low-titer CD-related antibodies), the exclusion of other causes of persistent villous atrophy, and phenotyping of the intestinal lymphocytic population aimed to confirm the presence (type 2) or absence (type 1) of a monoclonal rearrangement of T cell receptor. In all cases of type 2 RCD, it is essential to perform, at diagnosis, a computed tomography (CT) and/or magnetic resonance (MR) enterography followed by positron emission tomography (PET), capsule endoscopy, and enteroscopy in order to rule out the progression to intestinal lymphoma [152, 154]. Due to this risk, in subjects with a diagnosis of type 2 RCD, a capsule endoscopy has been recommended once a year at the follow-up [156]. From a therapeutic perspective, the management of type 1 RCD is based on immunosuppressive therapy containing steroids, azathioprine, 6-mercaptopurine, and methotrexate, whereas type 2 therapy is based on additional medications, including cyclosporine and chemotherapy such as cladribine and fludarabine associated with anti-CD52 monoclonal antibodies (alemtuzumab). Promising results have been recently reported by treating patients with anti-IL-15 antibodies (AMG-714). In certain cases, an autologous stem cell transplantation has been attempted with promising results [154–156].

### Intestinal lymphoma

The association between CD and cancers has been known for over 50 years [157] and a delayed diagnosis of CD exposes patients to an increased risk of developing neoplastic diseases [158]. In recent years, several studies have reported a growing incidence from 6 to 9 times higher than that of the general population for non-Hodgkin T cell intestinal lymphoma and, to a lesser extent, also B cell lymphoma [158]. In most cases, the development of intestinal lymphoma is preceded by type 2 RCD that develops into malignant disease in 33–52% of cases within 5 years from diagnosis. More rarely, intestinal lymphoma may develop from type 1 RCD, with a rate of 14% over 5 years [159]. Treatment in cases of CD-related intestinal lymphoma involves chemotherapy, i.e., high-dose ifosfamide, epirubicin, and etoposide methotrexate, followed by autologous stem cell transplantation. If lymphoma includes an elevated expression of CD30 (> 80% of the neoplasm) it is possible to use biologic therapy with anti-CD30 associated with monomethyl auristatin E (brentuximab vedotin) and a chemotherapy regimen containing cyclophosphamide–doxorubicin–prednisone followed by autologous stem cell transplantation [159]. Recent data indicate that NKp46, a NK receptor expressed by lymphocytes, can be a biomarker as well as a possible therapeutic target for T cell lymphoproliferative diseases, i.e., type 2 RCD and enteropathy-associated T cell lymphoma [160].

### Small bowel adenocarcinoma

Small bowel adenocarcinoma is an extremely rare cancer in the general population (5.7 cases/1,000,000 people per year) but it is much more common in patients with CD (odds ratio reported in the literature ranges between 4.3 to 60.0), usually being detectable in the jejunum [161]. Compared to lymphomas, small bowel adenocarcinoma is rare, although increasingly detectable in the clinic. Nowadays, however, the diagnosis of this cancer occurs together with CD. Unlike intestinal lymphoma, the small bowel adenocarcinoma is not preceded by RCD and occurs more commonly in female patients [150]. The onset of a sudden intestinal (sub)/occlusion and/or anemia, particularly in patients with a late diagnosis of CD and patients who have been following a GFD for a short period of time, are clinical features suggestive of an underlying small bowel adenocarcinoma. A thorough diagnostic work-up is mandatory and requires a wide array of imaging tests (e.g., CT/MR-enterography, PET, capsule endoscopy, and enteroscopy) [162].

## Follow-up for CD in adults

A well-defined follow-up strategy should be agreed by physicians and patients once CD has been diagnosed. Usually, the first follow-up visit is planned within 6 months from diagnosis and then every 12–24 months (every 3–6 months if complications occur) is adequate to confirm compliance with the GFD, rule out the onset of autoimmune diseases and metabolic changes, and, most importantly, to allow for the early diagnosis of any complications [163]. Patients should undergo a consultation with a dietician and follow-up blood tests including complete blood count, anti-tTG IgA (or IgG in case of IgA deficiency), thyroid stimulating hormone, anti-thyroidperoxidase, anti-thyroglobulin, ferritin, folate, vitamin D3, transaminases, and a metabolic profile [163]. The first follow-up should include a screening of antinuclear antibodies and non-organ-specific autoantibodies in order to rule out the presence of markers predictive of autoimmune diseases associated with CD. Should the antinuclear antibodies test reveal a high titer along with extractable nuclear antigen antibody positivity, this information might be useful to investigate for other autoimmune CD-associated disorders, e.g., primary biliary cholangitis and Sjogren syndrome [2]. In adults, a bone density scan should be performed after 12–18 months of a GFD and repeated regularly only if abnormal or in case of other indications. Subjects with osteopenia should be treated with supplements containing calcium and vitamin D, while possible treatment with bisphosphonates should be considered in cases of osteoporosis. Body weight increase may occur as a consequence of an excessive consumption of dietary products high in vegetable fats (colza, palm, and coconut oil) commonly present in GFD [164]. Therefore, nutritional counselling is advisable to prevent metabolic complications, including liver steatosis, during follow-up. On the other hand, patients who are starting GFD should be tested with an abdominal ultrasound to exclude spleen abnormality (i.e., hyposplenism) [165].

Notwithstanding a strict GFD, CD patients may experience abdominal symptoms ascribable to IBS in 30–50% of cases; these symptoms may respond to dietary recommendations (e.g., reduction of insoluble fiber intake or fermentable oligosaccharides, disaccharides, monosaccharides, and polyols) as well as symptomatic drug therapy [166].

A self-adapted GFD, without the support of a nutritionist, can cause vitamin and trace metal deficiency, which should be supplemented if needed, particularly when patients report the onset of asthenia [167]. Additionally, constipation, which can be associated with a GFD, requires appropriate management based on non-irritant (e.g., osmotic) laxatives [168].

Should a CD-related complication occur, follow-up visits should be more frequent, i.e., every 3–6 months [156]. In these circumstances, in addition to standard tests (as previously listed), protein electrophoresis, lactate dehydrogenase, and beta-2 microglobulin testing should be included. Upper endoscopy should be performed (with new duodenal biopsies) along with abdominal ultrasound, as well as CT/MR-enterography, PET, capsule endoscopy, and enteroscopy [154–156].

Physicians may consider (even if not recommended by current guidelines) performing a follow-up duodenal biopsy in adults in order to check the regrowth of villi in patients on a GFD, keeping in mind that the average time to the restitutio ad integrum of the villi could take up to 3 years. A second duodenal biopsy after GFD should be recommended only in those patients with persisting symptoms and demonstrable laboratory deficiencies of micronutrients [133].

Finally, GIP assessment, a controversial test still awaiting further validation, can be performed on stool samples and may be useful for monitoring the adherence to a GFD [48].

## Follow-up for CD in children

Currently, the follow-up of CD in children is lacking standardized evidence-based recommendations [169]. Children with CD should be followed up after 6 months from diagnosis and then every year in order to check symptomatic improvement, adherence to GFD, quality of life, and progressive normalization of CD-related antibodies. Laboratory tests and biochemical evaluation is crucial in these patients and should be tailored on case-by-case basis. As for adults, autoimmune thyroiditis should always be screened. Duodenal biopsy monitoring is unnecessary after a GFD has been instituted. However, should the patient have no or partial clinical response to gluten withdrawal, a careful assessment should be recommended to rule out inadvertent gluten ingestion or poor adherence to a GFD. Furthermore, in this subset of poorly responsive patients, a duodenal histopathology is advisable [119, 169]. At variance to adults, children hardly ever develop complications, indeed only a few case reports of refractory CD have been reported [170].

## Diet and new treatments

Currently, the only effective treatment available for CD is a strict GFD for life since it leads to the resolution of intestinal and extraintestinal symptoms, negativity of autoantibodies, and the regrowth of the intestinal villi. In addition, the diet offers a partial protective effect towards several complications. However, these crucial advantages are accompanied by some disadvantages, including a negative impact on quality of life, psychological problems, fear of involuntary/inadvertent contamination with gluten (as demonstrated in multicenter GIP studies) [48], possible vitamin and mineral deficiencies, metabolic syndrome, an increased cardiovascular risk, and often severe constipation [171–173]. Most of these CD-related drawbacks can be overcome by instructing the patient about the risks of an uncontrolled gluten-free regimen and by providing nutritional recommendations by a dietician with experience in CD. From a psychological perspective, the support a psychologist could be highly useful in accepting the disease [174].

Due to the relevant burden induced by gluten withdrawal with consequent worsening of quality of life, about 40% of CD patients are unsatisfied with their alimentary regimen and they would be keen to explore alternative treatments [175]. In recent years, researchers have attempted to meet the requests of CD patients seeking therapies different from diet [176]. Clinical trials are currently in progress, but only few have reached later clinical trial phases, namely those with larazotide acetate and gluten-specific proteases from a bacterial mix (ALV003) [177–180]. Larazotide acetate is a zonulin antagonist blocking tight junction disassembly, thereby limiting gluten crossing a permeable intestinal mucosal barrier [177]. Larazotide has shown efficacy in gluten-related symptom control rather than in restoring complete epithelial barrier integrity and preventing gluten from crossing the mucosal lining [177]. Taken together, the data so far published indicate that larazotide may be beneficial in allowing patients to tolerate minimal amounts of gluten such as those derived from inadvertent ingestion or probably for ‘gluten-free holidays’, i.e., a short period during which patients are allowed to eat a minimal amount of gluten. ALV003 targets gluten and degrades it into small fragments in the stomach before they pass into the duodenum [178]. This strategy has also been demonstrated to be able to ‘digest’ only small quantities of gluten and thus would be effective against contamination but not to protect patients from the effects driven by large quantities of gluten [178]. However, a recent phase 2b study by Murray et al. [180] showed that ALV003 (or latiglutenase) did not improve histologic and symptoms scores in 494 CD patients with moderate to severe symptoms versus placebo. IL-15 monoclonal antibodies (AMG 714) are being investigated in phase 2 studies in both gluten challenge and RCD type II patients, but additional safety studies are needed for the acquisition and competition of the license. Finally, vaccination (Nexvax2) is another possible therapeutic strategy aimed at desensitizing patients with CD to gliadin peptides. Although abdominal pain and vomiting were major side effects, the trial passed phase 1. Vaccines could represent a definitive cure for CD should data show actual efficacy [181].

### Can CD be prevented?

Several retrospective studies have suggested that breastfeeding, modality of delivery, and time of gluten introduction in the diet of infants at risk for CD may affect the incidence of the disease. However, the data supporting the role of these factors in the risk of developing CD is limited by their retrospective design and have been criticized by alternative interpretations [182–184]. Two recent landmark studies [19, 21], which prospectively screened infants with a first-degree family member with CD from birth, found that CD develops quite early in life in this risk group, demonstrating that early environmental factors may be crucial in the development of CD. However, these studies failed to identify possible targets to prevent CD, leading to the gut microbiota as the key element to scrutinize for possible innovative preventive strategies. In this line, viral (e.g., rotavirus) GI infections may potentiate subsequent development of CD. Thus, rotavirus vaccination seems to significantly decrease the risk of CD, in particular among children with early (before 6 months of age) gluten exposure [185]. The ongoing Celiac Disease Genomic, Environment, Microbiome, and Metabolomic study has been designed to identify potential primary prevention targets by establishing microbiome, metabolomic, and/or environmental factors responsible for loss of gluten tolerance, thus switching genetic predisposition to clinical outcome [186].

## Conclusions

Although there has been a substantial increase in the number of CD diagnoses over the last 30 years, many patients remain undiagnosed [187]. The flow-chart for identifying CD in adults must always include both serology and intestinal biopsy, whereas genetics should be performed only in selected cases. Diagnostic criteria should help physicians in avoiding misdiagnosis and missing cases of CD (i.e., seronegative patients with classic symptoms not undergoing biopsy) and preserve people from an unjustified GFD. The treatment for CD is still primarily a GFD, which requires significant patient education, motivation, and follow-up. Slow response occurs frequently, particularly in people diagnosed in adulthood. Persistent or recurring symptoms should lead to a review of the patient’s original diagnosis, exclude alternative diagnoses, evaluation of GFD quality, and serologic testing as well as histological assessment in order to monitor disease activity. In addition, evaluation for disorders that could cause persistent symptoms and complications of CD, such as refractory CD or lymphoma, should be pursued. The future opens to new therapeutic and preventive strategies, which are expected to improve the patient’s quality of life and pave the way to a definitive cure for this old disease.

Box 1 Causes for the increased number of intraepithelial lymphocytes in the intestinal mucosa with normal villous architecture Potential celiac disease

Non-celiac gluten sensitivity

Food allergies (cereals, milk proteins, soy derivatives, fish, rice, chicken)

Infectious (viral enteritis, Giardia, Cryptosporidium, *Helicobacter pylori*)

Bacterial contamination of the small intestine

Drugs (e.g., non-steroidal anti-inflammatory drugs)

Immune system diseases (Hashimoto’s thyroiditis, rheumatoid arthritis, systemic erythematosus lupus, type 1 diabetes mellitus, autoimmune enteropathy)

Common variable immune deficiency

Chronic inflammatory intestinal diseases (Crohn’s disease, ulcerative colitis)

Lymphocytic colitis

## Data Availability

None.

## Abbreviations

ACDCs: Associated celiac disease conditions
CD: Celiac disease
CT: Computed tomography
DGP: Deamidated gliadin peptides antibodies
EmA: Anti-endomysial antibodies
ESPGHAN: European Society for Paediatric Gastroenterology Hepatology and Nutrition
GFD: Gluten-free diet
GI: Gastrointestinal
GIP: Gluten immunogenic peptides
HLA: Human leukocyte antigen
IBS: Irritable bowel syndrome
IELs: Intraepithelial lymphocytes
IL: Interleukin
MR: Magnetic resonance
NK: Natural killer
OACD: Ongoing active celiac disease
PET: Positron emission tomography
RCD: Refractory celiac disease
TG2: Transglutaminase 2
tTG: tissue transglutaminase
